# Phthisis as Related to Syphilis and Scrofula

**Published:** 1873-07

**Authors:** H. C. Hand

**Affiliations:** St. Paul


*Phthisis as related to Syphilis and Scrofula.* Prize Essay by H. C. HAND, M.D., of St. Paul. (From Transactions of the Minnesota State Medical Society, 1873.)

A number of writers and lecturers, men of authority in the medical profession, have at various times advanced an idea that a relation existed between some cases of phthisis and syphilis, or, as most of them have worded it, "between syphilis and tuberculosis of the lungs," the sharp distinction between tuberculous and non-tuberculous phthisis having become but recently generally recognized.

Scrofula and tuberculosis were also once described as not essentially differing from each other, but of late have been more or less separated.

Phthisis is no longer viewed as necessarily, or even generally tuberculous; and any relation of syphilis to phthisis has been scarcely more than hinted at, certainly not proved, if any steps, indeed, have been taken towards the proof. In this obscure status of affairs the following problems present themselves:

1. Does syphilis ever produce phthisis, and if so, is it a frequent cause?

2. Granted that it does, in what way, and what form, the tuberculous or pneumonic?

3. Are tuberculosis and scrofula closely allied, or even identical?

4. What connection, if any, has syphilis with scrofula and tuberculosis?

In the spring of 1870, beside many equivocal cases which were excluded from his note book, the writer had access to and partial care of fifty-five cases of constitutional syphilis; these were *all* examined as to the condition of their lungs, and the result is given in the following table. With a single exception, a case of chronic pleurisy (No. 27), the lung troubles have a history of having commenced since the initial sore, in most instances since the appearance of constitutional manifestations.

CASES OF SYPHILIS TABULATED WITH REFERENCE TO CONDITION OF LUNGS.

| No | Time since Appearance of Chancre. | Stage. | Family History. | Condition of Lungs. |
|---|---|---|---|---|
| 1 | 9 mos. | Secondary. | | Normal. |
| 2 | 3 yrs. | Tertiary. | | Pleuritic Adhesions. |
| 3 | 3 mos. | Secondary. | | Normal. |
| 4 | 4 yrs. | Tertiary. | | " |
| 5 | 13 yrs. | " | | Pleuritic Adhesions. |
| 6 | 4 mos. | Secondary. | | Normal. |
| 7 | 7 yrs. | " | Healthy. | Phthisis. |
| 8 | 3 yrs. | Tertiary. | | Normal. |
| 9 | 4 yrs. | " | | Repeated Pleurisies. |
| 10 | 2 yrs. | Secondary. | | Advanced Acute Phthisis. |
| 11 | ........ | " | | Phthisis. |
| 12 | 15 mos. | Tertiary. | | Normal. |
| 13 | 1 yr. | Secondary. | One Sister Consump. | Phthisis. |
| 14 | 3 mos. | " | | Bronchitis. |
| 15 | 7 yrs. | Tertiary. | | Normal. |
| 16 | 2 yrs. | " | Mother Consumptive. | " [Phthisis. |
| 17 | 6 yrs. | " | | Pleuritic Adhesions and Incipient |
| 18 | 5 yrs. | " | Healthy. | Normal. |
| 19 | 2 mos. | Secondary. | | " |
| 20 | 15 mos. | " | | Phthisis; Pleurisies. |
| 21 | 1 yr. | Tertiary. | | Normal. |
| 22 | 6 yrs. | " | | Phthisis. |
| 23 | 5 mos. | Secondary. | Father & Mother Con. | " |
| 24 | 9 mos. | " | Mother & Brother Con. | " |
| 25 | 3 mos. | " | Healthy. | Acute Phthisis. |
| 26 | 10 mos. | " | " | Normal. |
| 27 | 2 yrs. | Tertiary. | | Pleuritic Adhesions. |
| 28 | 4 mos. | Secondary. | Healthy. | Acute Pleurisy. |
| 29 | 2 yrs. | Tertiary. | " | Acute Bronchitis. |
| 30 | 9 yrs. | " | " | Phthisis; Pleuritic Adhesions. |
| 31 | 4 yrs. | Secondary. | | Phthisis. |
| 32 | 20 yrs. | Tertiary. | | Phthisis; Pleuritic Adhesions. |
| 33 | 2 yrs. | " | | Normal. |
| 34 | 2 yrs. | " | | Phthisis. |
| 35 | 10 yrs. | Secondary. | Healthy. | " |
| 36 | 1 yr. | Tertiary. | Mother Consumptive. | Pleuritic Adhesions. |
| 37 | 8 yrs. | " | | Normal. |
| 38 | 5 yrs. | " | | Phthisis; Pleuritic Adhesions. |
| 39 | 7 yrs. | " | | " " " |
| 40 | 1 yr. | Secondary. | | Normal. |
| 41 | 15 mos. | Tertiary. | | Phthisis. |
| 42 | 5 yrs. | " | | " |
| 43 | 3 mos. | Secondary. | | Acute Bronchitis. |
| 44 | 12 yrs. | Tertiary. | | Chronic Bronchitis. |
| 45 | 8 yrs. | " | | Normal. |
| 46 | 5 yrs. | " | | Emphysema. |
| 47 | 4 mos. | Secondary. | | Phthisis. |
| 48 | 3 yrs. | Tertiary. | | Normal. |
| 49 | 4 yrs. | " | Very Consumptive. | Phthisis. |
| 50 | 1 yr. | " | | Chronic Pneumonia. |
| 51 | 1 yr. | Secondary. | | Phthisis. |
| 52 | 14 mos. | Tertiary. | | Normal. |
| 53 | 1 yr. | Secondary. | | Phthisis. |
| 54 | 8 yrs. | Tertiary. | | Phthisis; Pleuritic Adhesions. |
| 55 | 14 yrs. | " | | Normal. |

It may be noticed that several of the cases, in which years have elapsed since the contraction of the chancre, are marked as secondary. By this is meant that no tertiary symptoms have appeared, not necessarily that the secondary still persist. In like manner active developments of tertiary lesions were not always present in the tertiary list, sometimes only their indelible footprints.

In seven of the above cases there was a coincidence of pleuritic adhesions and phthisis, the former either having ante-dated the latter, or being so extensive as to be incapable of being viewed as a sequence of it. These seven cases will account for the excess of diseases mentioned below over the number of patients.

In the fifty-five unselected syphilitic patients but twenty were found whose lungs were entirely sound, while there were twenty-four with phthisis in a more or less advanced stage. Twenty-four in fifty-five, forty-three per cent.! Surely a larger per cent. than is found in non-syphilitics, even taking into account the class of paupers among whom the observations were made. Here, then, is the first fact learned from the table, and we are now reasoning only from the table such as it is, although we might wish it fuller, larger, and more conclusive. *Phthisis is more frequent in syphilitics than in non-syphilitics.*

The only other lesion frequently noted was pleuritic adhesion in ten cases among the tertiary syphilitics, while in the secondary class no chronic pleurisies were found, but two cases of acute.

Let us review a few cases in detail in which a pulmonary trouble was superadded to syphilis, that we may attempt to gain some additional light from them.

I. Jane T., whose case was fully reported to the Society at its last Annual Meeting and published in the last volume of Transactions. While syphilis was running in her system a rapid and malignant course she was attacked by capillary bronchitis which led to lobular solidifications in the upper and lower lobes of both lungs. These solidifications were possessed of great firmness, and, although six months had elapsed between the date of their formation and the fatal termination, no attempt at softening was anywhere found.

II. Adam Jordan; æt. 24; colored; native of Alabama; had two chancres in March, 1869, and a non-suppurating bubo on each side. In the following July the lymphatic glands of neck began to swell, and in the fall a lichenous eruption covered the body. During April, May, and June, 1870, he was under observation, having the dark traces of his former eruption still remaining, and a fresh tuberculoid syphilide. The glands of the neck on each side, especially those below ramus of jaw, were enormously swollen, indolent and hard, the mass being larger that one's double fist. His condition remained essentially the same until October, 1870, when he slept on the floor of the ward and was attacked by pneumonia, of which he died at the end of six days.

*Autopsy;* 10 hours after death. Skin maculated. Neck slightly smaller than six months before. Broncial glands greatly enlarged, grayish black, and of diminished consistence. Lungs: posterior portion of both, and apex of one, in a state of gray hepatization, being of a uniform gray color, heavier than water, not crepitating on pressure and readily breaking under the finger; pus exudes from the cut ends of the small bronchial tubes. In those portions of the lungs comparatively free from disease, separate and distinct lobules are found undergoing the same process as above. No tubercle in either lung.

Spleen: triple or quadruple its normal size, dark red, firm, and nodulated by numerous, superficial and deep, yellow deposits, varying in size from a pin's head to a small pea. Splenic tissue around these deposits firmer than elsewhere. Liver and kidneys normal.

The salient points of the above case are, the cheesy degeneration of the cervical lymphatics, some of which were ulcerated and discharging externally, the embolic patches in the spleen, and the acute lobular pneumonia.

III. Thomas G., æt. 35, a native of Ireland, and of intemperate habits, having no hereditary taint, and previously healthy, in Dec., 1869, after having been employed in the water lifting lumber for two months, noticed his legs becoming stiff and painful, but not swollen. He had palpitation of the heart, stitches in his side, and a short dry cough. The stitches in side continued, as well as the cough, which after five months became attended with copious purulent expectoration and a little blood.

October 25, 1870, the following note was taken. He denies ever having had a chancre, but says that one year ago spots appeared on the skin, the earth-colored stains of which remain; he has slight general adenitis; sternal tenderness; his throat is congested and the seat of some white cicatrices; he has stinging, burning pains in palms of hands and soles of feet; and *had* severe nocturnal pain in legs and darting pains in the forehead with flashes of light in the eyes, which have been greatly relieved by a fortnight's course of iodide of potassium. He is emaciated and feeble. The nails are clubbed. Heart palpitates on exertion, but is not increased in size nor the seat of any murmur. Lungs: percussion clearness is impaired all over the chest, and the respiration is harsh, with many large and small moist rales; over right lower lobe the percussion is dull, respiration feeble, and movements of chest wall diminished.

Dec. 13. The emaciation, prostration, and physical signs have been gradually increasing, within the past few days the abdomen has become distended both with gas and with serous effusion, and to-day death occurred.

*Autopsy;* 5 hours after death.

*Heart:* right side filled with soft, dark clots. The whole organ is small, pale and flabby; the connective tissue around its base is distended with serum, and of an amber color.

*Pleural cavities:* right, entirely obliterated by firm adhesions; left, free in portions.

*Lungs:* at apex of right, and at bases of both, are small portions of pulmonary tissue permeable to air; even these portions, however, are not free from a nodular deposit. All the remaining portions are firm, of a specific gravity equaling or exceeding that of water, of a grayish-green color, and scattered over with white granulations, which are clustered somewhat after the manner of grapes on their stem. In the centre of many of these granulations can be seen a minute point, either dark or bloody. By careful manipulation, a bristle can be introduced at these points and passed along the vessel, which, by tracing downward to the heart, is found to be a branch of the pulmonary artery. The lower lobe of right lung is greatly diminished in size by the encroachment of the solidified and enlarged upper lobe; the false membranes between it and diaphragm, and its own tissue, are the seat of softening, chessy masses. The apex of the left lung is filled with communicating cavities.

*Bronchial glands:* enlarged and firm.

*Peritoneum:* smooth and shining, its cavity contains one gallon of clear, straw-colored liquid, which, in a few minutes after removal, gelatinizes into large, soft clots.

*Liver:* normal in size, dark in color; capsule on upper surface of right lobe is white and thickened.

*Spleen:* normal in size, its capsule thickened.

From the history given by the patient, and the symptoms presented while under observation, little room is left for doubt that he was the subject of syphilis, and in connection with this we have seen that he had grayish-green lobular pneumonic solidifications, in some locations softening and forming cavities. The *character* of the granulations found scattered throughout the pneumonic tissue is worthy of being noticed here. From their gross appearance, and the fact that they clustered on and around minute arterial branches, the microscopical characters likewise supporting the view, the writer feels no hesitancy in pronouncing them tuberculous granulations; but from their insignificancy of number as compared to the extensive inflammatory lesions, and their pearly freshness and undegeneracy, he feels equally justified in viewing them as a secondary phenomenon, a complication of the phthisis.

IV. Emma S., æt. 18, of delicate frame, was born in a brothel, and at the age of 13 was forced by her mother into the same business that she herself was following.

In June, 1868, she suffered from a chancre, gonorrhœa and venereal warts; and in April, 1869, had the same troubles. In January, 1870, she renewed her gonorrhœa, and this had not subsided when she came under observation on the 1st of April,

1870. At this time her hair was thin and dry, the pharynx was congested, the supra-condyloid and post-cervical glands were enlarged, the lower piece of the sternum was tender, and she gave a history of repeated eruptions on the skin, and attacks of sore throat.

In September, 1869, she first noticed a cough, with pain in the chest, which has steadily grown worse. Feb. 1870, she commenced to expectorate freely. May 6, 1870.—All over the upper lobe of right lung the percussion is dull, the respiration is cavernous, and there are gurgling rales. Lower lobe of the same lung: dullness on percussion, harsh respiration, prolonged expiration, and large moist rales. Left side: clear on percussion, respiration puerile; at very apex of lung are a few moist crackles. She is weak, emaciated, has hectic fever, and is greatly racked and worried by her cough. On the 28th of the following August she died. At the *autopsy* the right lung was found reduced to nothing but a honey-comb of various-sized cavities. Left upper lobe the seat of many tuberculous deposits, and some cavities. Left lower lobe solidified, containing some tuberculous deposits. *No part of the lungs would float in water.* Pleuritic adhesions were abundant; the diaphragm was also adherent to the liver, and the false membranes were the seat of some miliary tubercles.

The history of this poor girl is duplicated in the writer's memory by at least half a dozen similar ones. Girls, thrown early on the town, have burned out their young vitality by unendurable excesses, have contracted syphilis, and before reaching the age of twenty-five have paid the death penalty by phthisis of rapid course. That the syphilis has borne a hand in the origin and progress of their ultimate destroyer, he feels that he cannot doubt,—still, a hand that is, perhaps, secondary in efficiency as compared with the necessary conditions of their lives, the excitement, dissipation, and excesses, the weariness of body, dejection of spirits, and anguish of soul to which they are daily subjected.

Need more be said, or more cases quoted, to make the proposition good, that *secondary syphilis predisposes the patient to acute inflammation of the respiratory organs?* Of the acute pleurisies there is no necessity of a fuller mention at present; not so the acute bronchitis, and the acute phthisis as connected with the acute bronchitis. The two cases of bronchitis in the table were of the larger tubes. Jane Thompson, (Case I,) had capillary bronchitis; and in more than one other case, of which we have no note preserved, the same tendency of bronchitis, occurring in syphilitics, to run into the finer tubes, has been noticed. In some cases, such as this, the effused products not being absorbed, are endowed with sufficient vitality to remain as firm, organized masses, becoming converted into solidified lobules. In another class of cases the effusion is that of an asthenic, a catarrhal pneumonia, and has no tendency to absorption, while it is only capable of very imperfect organization: as examples, Cases III and IV may be mentioned. The non-progressive and feebly vitalized lymph begins to degenerate almost as soon as fairly effused; in its degeneration and destruction the original elements of the lung tissue are involved, and a proper example of acute phthisis is the result. Be it observed that *post-mortem* few or no miliary tubercles may be found, and these few secondary, the most prominent feature being the cheesy masses, called tubercle by the older pathologists, but now deprived of the title, and viewed as a lymph of asthenia and of low vitality; by some called scrofulous lymph. From this disease, characterized by the rapid melting away of pulmonary structure, nothing can be more widely distinct than acute miliary tuberculosis of the lungs, in which death takes place by a direct blow at the life force before softening and local tissue destruction have scarcely had a chance to commence. It appears to the writer that not even in the chronic forms of phthisis is the distinction between tuberculous and non-tuberculous so well marked as here, for the secondary deposition of miliary tubercles around a chronic pneumonia, will often obscure the true nature of the primary lesion. But in acute tuberculosis, death, as a rule, occurs before softening has had time to commence; while in the inflammatory effusions which end in acute destructive phthisis, the depositions are copious instead of miliary, and their *immediate* tendency is to perish and liquefy, in their death implicating and destroying with great rapidity all the infiltrated and adjacent pulmonary tissue, so that after a few weeks' course a *post-mortem* examination will reveal the former site of the lungs occupied by a honey-comb of cavities, the tuberculæ of which are themselves infiltrated and softening to such an extent that perhaps not a single normal air vesicle can be found.

Again, it is not to be understood that any exclusive or essential causative connection is claimed between secondary syphilis and acute pneumonic phthisis; their occasional, or even frequent, co-existence and apparent relationship are merely pointed out. The depressing influence of syphilis, together with an increased tendency, during its secondary stage, of the lungs to be attacked by capillary bronchitis and catarrhal pneumonia, is the nearest to a causal agency that can now be claimed for the disease. These catarrhal inflammations are essentially asthenic, and leave products in the air vesicles which are both incapable of absorption and of more than a very low organization; consequently they soften, destroy the adjacent structures, and become very properly classed under the head of phthisis of the lungs. The appearances found *post-mortem* in the lungs of Jane Thompson, were those more or less common to capillary bronchitis and lobular pneumonia. From her history, and the physical signs observed, undoubtedly her trouble was originally capillary bronchitis, and this became subsequently the cause of the pneumonic—if you please—solidifications of the pulmonary lobules; to perplex ourselves further as to whether we shall use one name or the other in the classification of the disease with its lesions, would be useless waste of ink in drawing lines of distinction where no material difference exists.

Whilst it is not the purpose of the writer to make any dogmatical statements, it may be readily seen that the first two of the questions which were propounded at the outset of this essay have in his mind met with answers as follows:

1. Syphilis is quite a frequent cause of phthisis.
2. The form so produced is usually the pneumonic.

While the other two questions are included with less propriety in the consideration of the subject allotted for the prize essay, and therefore can claim only a brief notice, they are of such interest that the opportunity to say a few words concerning them will not be allowed to pass unimproved.

To the question as to whether tuberculosis and scrofulosis are closely allied, or even identical, there seems to be at present but the possibility of one answer. All the later pathologists have become convinced of the frequent succession of localized, and at times general, tuberculosis to scrofulous, cheesy degeneration. This opinion is based on the fact that tuberculosis of the internal organs has again and again been produced, under the observation of such men as Hoffmann, Lebert, Cohnheim, and others of the same stamp, by inoculation with cheesy detritus; and also by the frequent occurrence of miliary tubercles in the vicinity of cheesy depots in the lungs, lymphatic glands, etc.

Of those tubercles which are secondary to cheesy inflammations Rindfleisch speaks as follows: "The confusion of names and ideas which this distinction (between miliary tubercles and cheesy masses,) already founded by Reinhard, but strictly carried out by Virchow, has occasioned, is not decreased by the circumstance that in fact, cheesy inflammation and miliary tuberculosis very commonly occur side by side. The latest times have also brought very interesting disclosures on this point; namely, according to a series of investigations, which were started by Villemain, continued by Klebs and others, and brought to a certain conclusion by Cohnheim, the introduction of 'cheesy detritus' into the juices of an individual results in the occurrence of 'miliary tuberculosis.' It is, therefore, a matter of indifference whether the cheesy material is transferred by inoculation, or whether it arises in the organism itself. Accordingly, the smallest particles of the cheesy detritus would have to be regarded as a poison, which by direct irritation occasions the tuberculous new formation of certain constituents of the tissues. This much is certain, that the formation of tubercle is the expression of a commenced dyscrasia, a corruption of the juices, which in many cases diffuses itself from a point throughout the organism; while in others the dyscrasia is probably (?) already congenital."

That secondary miliary tubercles are produced by the absorption of cheesy particles, and their subsequent arrest in the form of emboli, seems, at first sight, the most probable explanation of the *modus operandi* of the cause under consideration: but such a theory is overthrown by the absence of occlusion of the vessels at the site of the tubercle, except rarely, and then as the result, not of an embolus, but from excessive tumefaction of the vascular walls.

That the infection, in some instances, is in no way conveyed by the vascular system, but is strictly a local contamination, is conclusively proved by a case related by Rindfleisch, in which a cheesy depot existed in one lung. Around this depot in the lung tissue was a brood of miliary tubercles. On the pulmonary pleura covering it there was a dense pavement of tubercles; *and on the costal pleura, opposing the pavement, miliary tubercles were scattered*, but in more moderate numbers.

This question, then, has answered itself, that there are certain differences of structure and progress between scrofulous lymph, or the "cheesy masses," and miliary tubercle, which forbid that they should be considered identical; while certain relationships of coexistence and causation force us to believe them to be very closely allied.

The same reasons which show tuberculosis to be allied to scrofula, with the same cogency prove it to be allied to all the other diseases which carry cheesy degeneration in their train. Let us mention typhoid fever with its cheesy depositions in the solitary follicles and Peyer's patches of the intestines, and the not infrequent cheesy degeneration of the enlarged mesenteric glands. Too often does tuberculosis of the lungs arise as a sequel of the fever, directly dependent for its origin on the cheesy detritus just mentioned.

Now, syphilis in its third stage is ever manifesting itself by gummy tumors which possess a strong inherent tendency to exhibit the cheesy metamorphosis, and when once that metamorphosis has occurred, the resemblance to the cheesy masses of scrofula is perfect, and they are equally liable to become the leaven from which miliary tubercles are propagated in various parts of the body. This, then, is the connection which syphilis seems to the writer to have with scrofula and tuberculosis. A relation, be it observed, which only distinctly shows itself in the tertiary stage of syphilis. A stage in which every thinking mind must have reflected on the exceedingly uncertain boundary which exists between the manifestations of syphilis and scrofula. While in hereditary syphilis we are sure still graver doubts as to the propriety of attempting to separate the two diseases have arisen; and the sins of the parent have been traced in the snuffling nose or enlarged lymphatic glands of the wasted scrofulous child, almost as certainly as in the palmar and plantar pemphigus of early infancy.

The following extracts from a case published in the *Boston Medical and Surgical Journal*, for July 18, 1872, illustrate well the coexistence and interdependence of the phenomena of the diseases under consideration.

G. F., a colored laborer, having six years previously contracted the primary sore, was admitted to the Boston City Hospital. At the date of admission there was a sinus over the junction of coronal and sagittal sutures, discharging thin pus. This sinus led to necrosed bone, of which three pieces, each the size of a split pea, were removed. In the beginning of the second month of treatment a large cold abscess over the biceps of the arm was opened. A month later the right pleural cavity was found to be filled with liquid, giving rise to harassing cough, and sleeplessness from dyspnœa. The pulse continued above 100, and the appetite was greatly impaired. After a month the patient was convalescent, so that he walked about the ward. Soon, however, there followed extreme debility and diarrhœa; and the pulse rose to 136. The patient progressively sank, and at the end of four months from the date of entrance to hospital, died.

At the *autopsy* the following appearances were observed: In the *skull*, to the left of the median line, and a little anterior to the vertex, was an opening, one inch long by three-fourths of an inch wide, through which the dura mater could be seen. The dura mater was thickened, and slightly adherent to the pia mater, and the longitudinal sinus was somewhat constricted by this thickening.

The *tenth rib*, near its sternal end, was enlarged and necrosed, while in the adjacent tissue there was a deposit of *cheesy material*, in size about equal to a pigeon's egg. The articulating surface of the head of the right humerus was *necrosed*, as also were the glenoid cavity and acromion process of the scapula.

Both *lungs* were adherent to the thoracic walls, and in each pleural cavity there was a considerable quantity of serum. Each lung showed *scattered* deposits of *miliary tubercle*. Some of the bronchial glands had undergone *caseous degeneration*.

The *pericardium* was adherent in all its extent, and, with the deposit of lymph and false membrane, measured from one-fourth to one-half an inch in thickness. The muscular structure of the heart was thin. The valves were normal.

The *thyroid* and *thymus glands* were hypertrophied; each lobe of the latter was an inch and three-fourths long, and showed *cheesy degeneration*.

The *liver* presented two large masses of *cheesy metamorphosis*

near its surface, and two of smaller size internally. Small, puckered spots on its surface indicated the location of former disease.

The *spleen* contained a great number of large and small, deep and superficial, spots of *caseous degeneration.* Both *kidneys* showed signs of commencing parenchymatous lesion. The *mesenteric glands* were enlarged. In the small intestine there were several small ulcerations, and near the cœcum an ulcer of larger size. The solitary glands in the immediate vicinity of the ulcerations contained *cheesy deposits.*

Another case may be given from the writer's note book. Andrew S. died in 1870, after having for years been reputed to be suffering from syphilis. The basis for such repute seems to have been a sound one, consisting in blotches on the face, a severe and protracted sore throat, an offensive breath, and a worn and haggard appearance.

In Jan., 1872, the following note was made as to the condition of a son, 14 years old. He appears to have been unhealthy from birth, and for the last two years has had lymphatic enlargements under the jaw, above the clavicles, and behind the ears, which have opened and remain open as large ulcers with smooth, red and glazed bottoms and undermined edges, or have healed and left white, puckered cicatrices. From one ear there has been for the same length of time a free discharge of pus. His eyes have occasionally been crossed. At the date of the note he was suffering with fever, a violent pain in the head, and delirium. The forehead was wrinkled, and the patient constantly moaned in such a way as to direct attention to the head. Although there was no paralysis of the eyes, tongue, or other part of the body, nor any irregularity of the pupils, the symptoms were such as to warrant the supposition of sub-acute meningitis, very possibly induced by incipient caries of the bones at the base of skull. Under the use of iodine and good food, however, he improved greatly, and at the end of ten months his intellect is as clear as before the attack, and all the other brain symptoms have subsided.

Another son of the same father, seven years old, is almost blind from the presence of leucomata on each cornea, left by the healing of obstinate ulcers. While, beginning with the sixth dorsal vertebra and extending to the tenth, there is a well-marked antero-posterior curvature of the spine.

Such examples might be almost indefinitely multiplied. Thus, Dr. Parry reports in the *Medical Times*, June 1, 1871, the autopsy of a colored boy of seven years. Cheesy masses, which appear to have been degenerated syphilitic gummata, were found in the spleen and on the inside and outside of the cranium. The bones of base skull were carious. The bodies of the third and fourth dorsal vertebræ were entirely destroyed by caries and their place filled by cheesy material. The cervical lymphatic glands were enlarged, the bronchial glands had grown to the size of a lemon, and were rapidly undergoing the cheesy metamorphosis. In the lungs, the spleen, and the membranes of the brain, were miliary tubercules.

That the views herein advocated are not too young to be true, is conclusively shown by the quotation given below from Benjamin Bell's work on the "Venereal." He says: "Where evident symptoms of scrofula have previously taken place, or where that disease obviously exists at the time, there is no great difficulty of convincing patients of any symptom of Lues Venerea with which they are attacked, being likely to partake of it; but they should also know that during the continuance of Lues Venerea, symptoms of scrofula frequently appear when that disease was not previously suspected to exist, and which might otherwise have never taken place. Of this I have met with many instances, where a scrofulous taint, which had till then remained concealed, broke out at once with much more violence on the system being attacked with Lues Venerea."

Niemeyer in his "Clinical Lectures on Pulmonary Phthisis," has said that "tuberculosis, in most cases, is a secondary disease, arising in a manner not known to us through the influence of caseous morbid products on the organism." The usual seat of such caseous morbid products is found to be in the lungs during the degeneration of chronic inflammatory deposits. In other cases we have to look for the *fons et origo* in the lymphatic glands, in carious bone, or, perhaps, in an exudation on the surface of the pleura or peritoneum.

When Niemeyer says that the worst thing that can happen to a phthisical patient is to become tuberculous, he utters the belief of all modern pathologists, and at the same time throws a strong light on the fact that tuberculosis is so often secondary. Why does it not occur in every case of phthisis which is in the stage of cheesy degeneration, as well as with every case of scrofulous lymphatics, and, indeed, whenever a cheesy nidus is found in any organ or structure of the body? It has been suggested, with a show of reason, that the increased growth of connective tissue around the caseous matter encysts it and prevents its exit. And this view is sustained by the fact, recognized since the time of Laennec, that a deposition of miliary tubercles often occurs a short time before death, when by reason of that softening and destruction of the lung which is very apt to occur in the last stages of the disease, the protecting barrier may be supposed to be broken down.

But what can be said of such an assertion as that recently made by Dr. Alfred Meadows, viz: that tuberculosis and scrofula are not identical, but in many respects *absolutely* dissimilar and to some extent *even antagonistic?* What, indeed, but that his experience must then have been widely different from that of the majority of medical practitioners, and with them the writer.

As any essay is manifestly more directly valuable to the reader if it point its arguments by showing how a practical application of its theories may be made; and as this is all the more true in Medicine because of the too frequent doubts in which the practitioner is left to grovel after the proper and rational mode of treatment, this humble attempt to set forth some of the truths of the pathology of the broad subject considered shall be closed by one or two brief suggestions as to treatment.

It is admitted on all hands that the natural tendency of tubercle is towards death, not only the local death of the part in which the tubercle is located, but finally the death of the individual who is its unfortunate bearer. So long as tuberculosis was supposed to be capable of being produced *only* by the tuberculous diathesis, all attempts directed to the prevention of the deposition were necessarily considered as futile; while those who, like Dr. Meadows, think the scrofulous diathesis to be antagonistic to the tuberculous, if consistent, would encourage the former to prevent the latter. But, if tuberculosis may be caused by some malign influence which cheesy deposits are capable of exerting on the system, then riddance of the system of these foci of disease becomes our rational course. And when they are situated near the exterior of the body, as in the superficial lymphatics, or are associated with carious bone, what means is equal to the well-directed knife for their prompt removal? On this principle rests the recommendation which has recently been made that chronic lymphatic tumors, instead of being trusted to the slow efforts of nature at absorption, should be extirpated whenever and wherever such a proceeding is practicable. And on the same principle the success attained in restoring to health those afflicted with caries of the head of the femur, and of other bones, by a judiciously early excision, is explained; rather than as formerly allowing nature to work her tedious way towards a cure, the attainment of which would probably never be reached, because either the patient would succumb to secondary tuberculosis, or to amyloid degeneration induced by the protracted suppuration, before the desired cure could be attained. It has been a common observation that scrofulous children are safer from the supervention of pulmonary phthisis if their cervical lymphatic tumors suppurate and remain as open sinuses. And simply because the caseous material is thus freely cast out of the body, instead of being pent up and allowed full opportunity to contaminate the system. On the fact of the beneficial influence of *open* lymphomata over the life and health of the scrofulous sufferer, is probably based the opinion of *antagonism* between scrofula and tuberculosis; while in reality it is a strong argument on the other side, because the scrofulous products are thus cast out of the organism and prevented from working its general contamination.

In an essay on Phthisis presented to a Society, the climate of whose State is held in so high repute as is that of Minnesota, it might naturally be expected that climatic cure would have been the theme. Such a theme was avoided because it occurs to the writer that enough words have already been spilled in lauding our climate. Facts must now be collected, and they can alone convince the world that what has been already said and written has been put forth in good faith.

No one conversant with the exhilarating, vitalizing character of Minnesota air can for a moment doubt that it is capable of rescuing from the grave the incipient consumptive, or him who is about to become a consumptive. Let the last paragraph of this paper, then, point the lesson that here may also be found a balm to heal the sorrows of the one who is reaping the late fruits of early transgression, and to mitigate the penalty of the child on whose innocent head is being visited the sin of the father.—*Northwestern Med. & Surg. Journal.*

